# Impact of Feeding Postbiotics and Paraprobiotics Produced From *Lactiplantibacillus plantarum* on Colon Mucosa Microbiota in Broiler Chickens

**DOI:** 10.3389/fvets.2022.859284

**Published:** 2022-03-29

**Authors:** Yohanna Danladi, Teck Chwen Loh, Hooi Ling Foo, Henny Akit, Nur Aida Md Tamrin, Azizi Mohammad Naeem

**Affiliations:** ^1^Department of Animal Science, Faculty of Agriculture, Universiti Putra Malaysia, Serdang, Malaysia; ^2^Institute of Tropical Agriculture and Food Security, Universiti Putra Malaysia, Serdang, Malaysia; ^3^Department of Bioprocess Technology, Faculty of Biotechnology and Biomolecular Sciences, Universiti Putra Malaysia, Serdang, Malaysia; ^4^Institute of Bioscience, Universiti Putra Malaysia, Serdang, Malaysia

**Keywords:** postbiotics, paraprobiotics, colon mucosa, microbiota, broiler

## Abstract

This study was conducted to evaluate the impact of feeding postbiotics and paraprobiotics produced from *Lactiplantibacillus plantarum* on colon mucosa microbiota in broiler chickens. In this study, 336 one-day-old COBB 500 chicks were randomly allotted to eight treatment groups and replicated six times with seven birds per replicate. The treatment included T1 (Negative control) = Basal diet, T2 (Positive control) = Basal diet + 0.01% oxytetracycline, T3 = Basal diet + 0.2% postbiotic TL1, T4 = Basal diet + 0.2% postbiotic RS5, T5 = Basal diet + 0.2% paraprobiotic RG11, T6 = Basal diet + 0.2% postbiotic RI11, T7 = Basal diet + 0.2% paraprobiotic RG14, and T8 = Basal diet + 0.2% paraprobiotic RI11. There were reported changes in the bacterial community using 16S rRNA sequencing of the colon mucosa. The results of the sequencing of 16S rRNA genes in the colon mucosa samples indicated that compared to birds fed the negative control diet, birds fed paraprobiotic RI11 diets were recorded to have a lower relative abundance of Proteobacteria, while those fed the positive control were recorded to have a higher proportion of Firmicutes. Also, lower *Enterococcus* was reported in paraprobiotic RI11, while the most abundant genus was *Bacteroides* in postbiotic TL1. This study revealed that supplementation of postbiotics and paraprobiotics in the diets of broilers demonstrated positive effects on the microbiota by supporting the increase of beneficial microbes like the *Firmicutes* while decreasing harmful microbes like the *Proteobacteria*. Therefore, this study has provided knowledge on the modification of chicken mucosa microbiota through the feeding of postbiotics and paraprobiotics.

## Introduction

The gastrointestinal tract (GIT) of chicken hosts a great microbial community, and its integrity play an important role in nutrient absorption, development of immunity, and disease resistance (1). When changes occur in the GIT microbiome, feed efficiency, productivity, and health of the birds can be influenced (2–4). The different sections of the GIT of chickens are heavily populated with complex microbiome (bacteria, fungi, Archaea, protozoa, and virus) dominated by bacteria (5). The gut microbiota can create a protective barrier by attaching to the epithelial walls of enterocytes, thereby reducing the possibility of colonising pathogenic bacteria (6).

Primarily, the commensal microbiota can benefit the host in terms of competitive exclusion of pathogens or non-indigenous microbes, immune stimulation and programming, and contributions to the host nutrition (7). In addition, they can stimulate the development of the immune system, including mucus layer, epithelial monolayer, intestinal immune cells (e.g., cytotoxic and helper T cells, immunoglobulin producing cells, and phagocytic cells), and the lamina propria (7–9). The gut mucus layer increases mucin secretion and epithelial cell turnover through the influence of the commensal microbiota. This helps keep the GIT lubricated while preventing microorganisms from taking over the intestinal epithelial cells of the host (1).

The microbiota in the hindgut (caeca and colon) can produce energy and nutrients like vitamins, amino acids, and short-chain fatty acids (SCFA) from the undigested feed, which are later available to the host (2, 7). The bacteriostatic properties of SCFA can destroy foodborne pathogens such as *Salmonella* sp. (10). Previous reports have already established that the pH of the colon becomes lower as a result of SCFA production, which inhibits the conversion of bile to secondary bile products (11).

Diets greatly impact the intestinal microbiome of chickens. Different constituents of diet that escape digestion and absorption by the host are being utilised as a substrate for growth by the bacteria (12). The diversity and composition of the gut microbiome in poultry are greatly impacted by diets (13).

The composition of the mucosa attached microbiota is influenced by several host factors such as the expression of specific adhesion sites on the enterocyte membrane, secretion of secretory immunoglobulins, and mucus production rate (1). It is well established that diet can alter luminal and mucosa-attached microbiota to influence gut health (3). The use of postbiotics as a replacement for antibiotics in broiler chicken diets has been well documented (14–17). Postbiotic has been defined as any factors resulting from the metabolic activity of a probiotic or any released molecules capable of conferring beneficial effects to the host directly or indirectly (18). Postbiotics are also known as non-viable bacterial products or metabolic by-products from probiotic microorganisms with biological activity in the host (18, 19). In contrast, the term paraprobiotics was coined to indicate the use of inactivated probiotic cells (non-viable) or probiotic cell fractions to confer health benefits to the host (20). Hence, paraprobiotics are also known as “inactivated probiotics” or ghost probiotics (20). The benefits of postbiotics include inhibiting pathogenic bacteria growth, leading to efficient nutrient utilisation and improvement in growth (21–23). On the other hand, the benefits provided to the host by paraprobiotics include modulation of the immune system, whereby the cell wall components may boost the immunological responses (24, 25).

Extensive studies with postbiotics effects on targeted caecum microbial population have been carried out (14, 17, 26, 27). However, the effect of postbiotics and paraprobiotics on the colon bacterial microbiota is yet unknown. Similarly, postbiotics and paraprobiotics affect the microbiota living in the outer mucosa layer, which differs from the caecal microbiome. Mucosa microbiota found within the outer mucous layer plays an important role in the intestine (28, 29). Therefore, the colon mucosal genome was studied using next-generation sequencing (16S rRNA). This study aimed to evaluate the impact of postbiotics and paraprobiotics on the colon mucosal microbiota in broiler chickens.

## Materials and Methods

### Postbiotic and Paraprobiotic Preparations

The active culture of *Lactiplantibacillus plantarum* strains was washed once with sterile 0.85% (w/v) NaCl (Merck, Darmstadt, Germany) solution and adjusted to 10^9^ CFU/ml to be used as a 10% (v/v) inoculum according to the method described by Mohamad et al. (30). Both postbiotics and paraprobiotics were prepared according to the method described by Ooi et al. (31) using de Man, Rogosa, and Sharpe (MRS) medium and incubated at 30°C for 24 h under anaerobic conditions. As for postbiotic preparation, cell-free supernatant was collected as postbiotics after centrifugation at 10,000 × *g* for 15 min at 4°C. The cell suspension of *L. plantarum* strains was frozen for 7 days at −30°C to produce paraprobiotics.

### Broiler Chicken and Management

A total of 336 day-old COBB 500 chicks (DOCs) were obtained from a commercial hatchery. The DOCs were randomly distributed to 8 dietary treatments managed in a closed house system. The house temperature was set at 33°C ± 1°C on day 1 and was gradually reduced to about 25°C ± 1°C by day 15. The average relative humidity ranged between 60 and 75%. Each treatment group was replicated six times with seven birds per replicate and was managed in a 120 × 120 cm (length × width) pen cage. The dietary treatment included T1 (Negative control) = Basal diet, T2 (Positive control) = Basal diet + 0.01% oxytetracycline, T3 = Basal diet + 0.2% TL1 postbiotic, T4 = Basal diet + 0.2% RS5 postbiotic, T5 = Basal diet + 0.2% RG11 paraprobiotic, T6 = Basal diet + 0.2% RI11 postbiotic, T7 = Basal diet + 0.2% RG11 paraprobiotic, and T8 = Basal diet + 0.2% RI11 paraprobiotic. The birds were vaccinated against Newcastle disease and infectious bronchitis disease (ND-IB) through eye drop at 7 and 21 days. The infectious bursal disease (IBD) vaccination was done on day 14 by eye drop. Water and feed were offered *ad libitum* until day 35. The starter and finisher diets (Tables 1, 2) were offered from days 0 to 21 and days 22 until 35 days of age, respectively. The experiment was undertaken based on the guidelines approved by the Institutional Animal Care and Use Committee of the Universiti Putra Malaysia (IACUC) with reference no. UPM/IACUC/AUP-R098/2018, which ensures that the care and use of animals for scientific purposes are humane and ethical.

**Table 1:** Nutrient composition of starter diets (days 1–21).

**Ingredients**	**Treatment diets**							
	**T1**	**T2**	**T3**	**T4**	**T5**	**T6**	**T7**	**T8**
Corn	47.50	47.49	47.20	47.20	47.20	47.20	47.20	47.20
Soybean meal	40.10	40.10	40.20	40.20	40.20	40.20	40.20	40.20
Wheat pollard	1.50	1.50	1.60	1.60	1.60	1.60	1.60	1.60
CPO	6.00	6.00	5.90	5.90	5.90	5.90	5.90	5.90
l-Lysine	0.50	0.50	0.50	0.50	0.50	0.50	0.50	0.50
dl-Methionine	0.50	0.50	0.50	0.50	0.50	0.50	0.50	0.50
Dicalcium phosphate	2.50	2.50	2.50	2.50	2.50	2.50	2.50	2.50
Calcium carbonate	0.45	0.45	0.45	0.45	0.45	0.45	0.45	0.45
Choline chloride	0.10	0.10	0.10	0.10	0.10	0.10	0.10	0.10
Salt	0.35	0.35	0.35	0.35	0.35	0.35	0.35	0.35
Mineral mix	0.15	0.15	0.15	0.15	0.15	0.15	0.15	0.15
Vitamin mix	0.15	0.15	0.15	0.15	0.15	0.15	0.15	0.15
Antioxidant	0.10	0.10	0.10	0.10	0.10	0.10	0.10	0.10
Toxin binder	0.10	0.10	0.10	0.10	0.10	0.10	0.10	0.10
Antibiotics	0.00	0.01	0.00	0.00	0.00	0.00	0.00	0.00
Postbiotic TL1	0.00	0.00	0.20	0.00	0.00	0.00	0.00	0.00
Postbiotic RS5	0.00	0.00	0.00	0.20	0.00	0.00	0.00	0.00
Paraprobiotic RG11	0.00	0.00	0.00	0.00	0.20	0.00	0.00	0.00
Postbiotic RI11	0.00	0.00	0.00	0.00	0.00	0.20	0.00	0.00
Paraprobiotic RG14	0.00	0.00	0.00	0.00	0.00	0.00	0.20	0.00
Paraprobiotic RI11	0.00	0.00	0.00	0.00	0.00	0.00	0.00	0.20
**Total**	**100**	**100**	**100**	**100**	**100**	**100**	**100**	**100**
**Calculated analysis**								
ME (kcal/kg)	3,215.70	3,215.70	3,201.40	3,201.40	3,201.40	3,201.40	3,201.40	3,201.40
Protein (%)	22.00	22.00	22.03	22.03	22.03	22.03	22.03	22.03
Fat (%)	7.99	7.99	7.88	7.88	7.88	7.88	7.88	7.88
Fibre (%)	4.11	4.11	4.12	4.12	4.12	4.12	4.12	4.12
Calcium (%)	1.08	1.08	1.08	1.08	1.08	1.08	1.08	1.08
Total Phos (%)	0.89	0.89	0.89	0.89	0.89	0.89	0.89	0.89
Avail. P (%)	0.48	0.48	0.48	0.48	0.48	0.48	0.48	0.48

*Negative control = T1 (Basal diet), positive control = T2 (Basal diet + 0.01% (w/w)Oxytetracycline), T3 = Basal diet + 0.2% (v/w) postbiotic TL1, T4 = Basal diet + 0.2% (v/w) Postbiotic RS5, T5 = Basal diet + 0.2% (v/w) paraprobiotic RG11, T6 = Basal diet + 0.2% (v/w) postbiotic RI11, T7 = Basal diet + 0.2% (v/w) paraprobiotic RG14, T8 = Basal diet + 0.2% (v/w) paraprobiotic RI11. Dicalcium phosphate 18%; vitamin premix provided per kilogram of diet: vitamin A 35.000 MIU; vitamin D3 9.000 MIU; vitamin E 90.000 g; vitamin K3 6.000 g; vitamin B1 7.000 g; vitamin B2 22.000 g; vitamin B6 12.000 g; vitamin B12 0.070 g; biotin 300.000 mg; pantothenic acid 35.000 g; nicotinic acid, 120.000 g; folic acid 3.000g; phytase 25,000.000 FTU. Mineral mix provided per kilogram of diet: Se 0.2 g, Cu 15 g, Fe 80 g, I 1 g, Mn 100 g, Na 1.5 g, Zn 80 g, K 4 g, and Co 0.25 g. Antioxidant contains butylated hydroxyanisole (BHA). Toxin binder contains natural hydrated sodium calcium aluminium silicates to reduce the exposure of feed to mycotoxins. Oxytetracycline (200 mg/kg, purity ≥ 64.7%, Y.S.P. Industries (M) SDN BHD). The diets were formulated using FeedLIVE International software (Nonthaburi, Thailand)*.

**Table 2:** Nutrient composition of finisher diets (days 22–35).

**Ingredients**	**Treatment diets**							
	**T1**	**T2**	**T3**	**T4**	**T5**	**T6**	**T7**	**T8**
Corn	51.60	51.59	51.60	51.60	51.60	51.60	51.60	51.60
Soybean meal	33.50	33.50	33.50	33.50	33.50	33.50	33.50	33.50
Wheat pollard	4.80	4.80	4.60	4.60	4.60	4.60	4.60	4.60
CPO	5.20	5.20	5.20	5.20	5.20	5.20	5.20	5.20
l-Lysine	0.50	0.50	0.50	0.50	0.50	0.50	0.50	0.50
dl-Methionine	0.50	0.50	0.50	0.50	0.50	0.50	0.50	0.50
Dicalcium phosphate	2.50	2.50	2.50	2.50	2.50	2.50	2.50	2.50
Calcium carbonate	0.45	0.45	0.45	0.45	0.45	0.45	0.45	0.45
Choline chloride	0.10	0.10	0.10	0.10	0.10	0.10	0.10	0.10
Salt	0.35	0.35	0.35	0.35	0.35	0.35	0.35	0.35
Mineral mix	0.15	0.15	0.15	0.15	0.15	0.15	0.15	0.15
Vitamin mix	0.15	0.15	0.15	0.15	0.15	0.15	0.15	0.15
Antioxidant	0.10	0.10	0.10	0.10	0.10	0.10	0.10	0.10
Toxin binder	0.10	0.10	0.10	0.10	0.10	0.10	0.10	0.10
Antibiotics	0.00	0.01	0.00	0.00	0.00	0.00	0.00	0.00
Postbiotic TL1	0.00	0.00	0.20	0.00	0.00	0.00	0.00	0.00
Postbiotic RS5	0.00	0.00	0.00	0.20	0.00	0.00	0.00	0.00
Paraprobiotic RG11	0.00	0.00	0.00	0.00	0.20	0.00	0.00	0.00
Postbiotic RI11	0.00	0.00	0.00	0.00	0.00	0.20	0.00	0.00
Paraprobiotic RG14	0.00	0.00	0.00	0.00	0.00	0.00	0.20	0.00
Paraprobiotic RI11	0.00	0.00	0.00	0.00	0.00	0.00	0.00	0.20
**Total**	**100**	**100**	**100**	**100**	**100**	**100**	**100**	**100**
**Calculated analysis**								
ME (kcal/kg)	3,180.83	3,180.83	3,176.68	3,176.68	3,176.68	3,176.68	3,176.68	3,176.68
Protein (%)	19.92	19.92	19.89	19.89	19.89	19.89	19.89	19.89
Fat (%)	7.29	7.29	7.29	7.29	7.29	7.29	7.29	7.29
Fibre (%)	4.01	4.01	3.99	3.99	3.99	3.99	3.99	3.99
Calcium (%)	1.06	1.06	1.06	1.06	1.06	1.06	1.06	1.06
Total Phos (%)	0.89	0.89	0.88	0.88	0.88	0.88	0.88	0.88
Avail. P (%)	0.48	0.48	0.48	0.48	0.48	0.48	0.48	0.48

*Negative control = T1 (Basal diet), positive control = T2 [Basal diet + 0.01% (w/w) Oxytetracycline], T3 = Basal diet + 0.2% (v/w) postbiotic TL1, T4 = Basal diet + 0.2% (v/w) Postbiotic RS5, T5 = Basal diet + 0.2% (v/w) paraprobiotic RG11, T6 = Basal diet + 0.2% (v/w) postbiotic RI11, T7 = Basal diet + 0.2% (v/w) paraprobiotic RG14, and T8 = Basal diet + 0.2% (v/w) paraprobiotic RI11. Dicalcium phosphate 18%; vitamin premix provided per kilogram of diet: vitamin A 35.000 MIU; vitamin D3 9.000 MIU; vitamin E 90.000 g; vitamin K3 6.000 g; vitamin B1 7.000 g; vitamin B2 22.000 g; vitamin B6 12.000 g; vitamin B12 0.070 g; biotin 300.000 mg; pantothenic acid 35.000 g; nicotinic acid, 120.000 g; folic acid 3.000g; phytase 25,000.000 FTU. Mineral mix provided per kilogram of diet: Se 0.2 g, Cu 15 g, Fe 80 g, I 1 g, Mn 100 g, Na 1.5 g, Zn 80 g, K 4 g, and Co 0.25 g. Antioxidant contains butylated hydroxyanisole (BHA). Toxin binder contains natural hydrated sodium calcium aluminium silicates to reduce the exposure of feed to mycotoxins. Oxytetracycline (200 mg/kg, purity ≥ 64.7%, Y.S.P. Industries (M) SDN BHD). The diets were formulated using FeedLIVE International software (Nonthaburi, Thailand)*.

### Mucosa Bacterial Metagenomic DNA

#### Sample Preparation

At the end of day 35 of the experiment, six chickens were randomly selected from each treatment. The chickens were slaughtered, and the mucosal scrapings from the colon were collected, quickly frozen, and later stored at −80°C until the time for analysis.

#### Bacterial Genomic DNA Extraction

The bacterial genomic DNA (gDNA) was extracted from the colonic mucosa samples using the NucleoSpin® DNA stool kit (Macherey-Nagel, GmbH and Co. KG, Düren, Germany). Approximately 200 mg of frozen colonic mucosa samples was lysed in ST1 buffer, the lysate was filtered using the NucleoSpin® Inhibitor Removal column, and Buffer ST2 was added to precipitate contaminants. Buffer ST3 was added to adjust the binding conditions, and the NucleoSpin® DNA Stool column was used to bind the DNA. The NucleoSpin® DNA Stool column was washed in four steps using buffers ST3, ST4, and ST5. Buffer SE was used to elute the DNA after the washing steps. DNA quality was verified using a NanoDrop 2000 spectrophotometer (Thermo Scientific, Wilmington, DE, USA) with a concentration (260/280 nm ratio absorbance) of extracted DNA.

#### 16S RRNA Sequencing of Colon Mucosa Microbiota

##### Bacterial 16S V3–V4 Amplicon Sequencing

**Figure 1:**
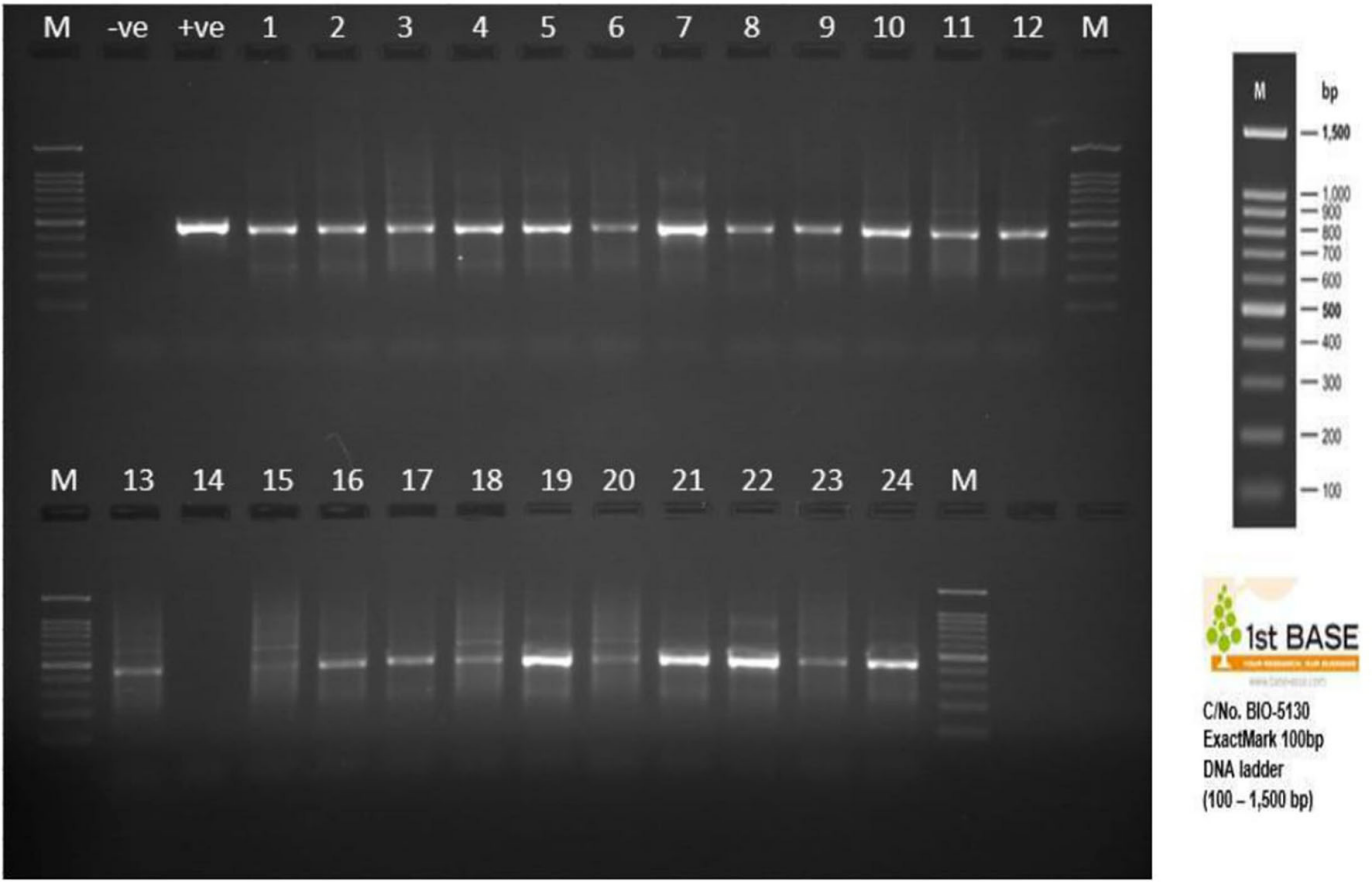
Bacterial gDNA fluorometric quantification.

Twenty-four (24) purified gDNAs were sent to Apical Scientific Laboratory, Sdn Bhd, Seri Kembangan, Malaysia, for the sequencing. The quality of the purified DNAs was first monitored on 1% Tris–acetate–EDTA (TAE) agarose gel. The concentration of DNA was measured using a spectrophotometer (Implen NanoPhotometer® N60/N50) and fluorometric quantification using iQuant™ Broad Range dsDNA Quantification Kit (Figure 1).

The purified gDNA that passed DNA sample QC was amplified using locus-specific sequence primers: 16S V3–V4 forward: CCTACGGGNGGCWGCAG, 16S V3–V4 reverse: GACTACHVGGGTATCTAATCC. All the PCRs were carried out with REDiant 2 × PCR Master Mix (1st BASE). Library preparations were done in two stages. The first stage of the PCR of bacterial 16S rRNA gene of the selected regions (16S V3–V4) was amplified using locus-specific sequence primers with overhang adapters, as follows:

Forward overhang: 5′TCGTCGGCAGCGTCAGATGTGTATAAGAGACAG-[locus-specific sequence]

Reverse overhang: 5′GTCTCGTGGGCTCGGAGATGTGTATAAGAGACAG-[locus-specific sequence]. All the PCRs were done using KOD, Multi & Epi® (Toyobo, Osaka, Japan).

At the second stage of PCR, dual indices were attached to the amplicon PCR using Illumina Nextera XT Index Kit v2 following the manufacturer's protocols. The quality of the libraries was measured using Agilent Bioanalyzer 2100 System by Agilent DNA 1000 Kit and fluorometric quantification by Helixyte Green™ Quantifying Reagent.

The libraries were normalised and pooled according to the protocol recommended by Illumina and proceeded to sequence using the MiSeq platform using 300 PE.

A phylogenetic tree was created using a combination of Multiple Alignment using Fast Fourier Transform (MAFFT) and FastTree algorithms. The MAFFT algorithms were used to construct a multiple sequence alignment (MSA), which was then passed to FastTree to construct a phylogenetic tree based on maximum-likelihood nearest-neighbour interchanges (NNIs). In addition, FastTree utilises the CAT estimation. It uses heuristics to restrict the search for a better phylogenetic tree and estimates a rate of evolution for each site at lower memory consumption and faster inference times.

##### Data Analysis

The sequence adapters and low-quality reads were removed from the paired-end reads before the first 200,000 raw reads were extracted using BBTools. Then, the forward and reverse reads were merged using QIIME. DADA2 pipeline (https://benjjneb.github.io/dada2/) was used to remove and correct error reads and to remove low-quality regions and chimeric errors. The resulting data were in the form of amplicon sequence variant (ASV) and was used in the next steps accordingly. The taxonomic classification was done using scikit-learn (https://scikit-learn.org/stable/) and naive Bayes classifier against database SILVA (release 132).

It is truly common in amplicon sequencing to involve a portion of the 16S rRNA gene or 18S gene, where the sequences are classified taxonomically. However, this involves few software and pipelines. The DADA2 pipeline comes with a naive Bayesian classifier that can classify large sequences across multiple ranks—from kingdom to genus—and provide an output in the form of taxonomy assignments with bootstrap confidence. It compares a set of taxonomically assigned sequences provided from formatted reference fasta files databases such as SILVA for ribosomal rRNA database and make individual taxonomic assignment (32). SILVA database (Release 132) was used to analyse the sequence similarity within the ASV reads with recommended parameters at a 97% similarity threshold (33).

##### Bioinformatic Analyses

Sequencing the region in 16S rRNA was done with a paired-end (PE) Illumina MiSeq platform that generates 300-bp raw reads. Sequence adapters and low-quality reads were removed from the raw reads using BBDuk (version 38.76). The raw reads are aligned and merged using QIIME2 (version 2019.10). The Divisive Amplicon Denoising Algorithm 2 (DADA2) pipeline (version 1.14) was used to denoise as an attempt to remove and correct error reads and to remove low-quality regions and chimeric errors to obtain ASV (34). DADA2 pipeline was used in this analysis to substitute the traditional operational taxonomic unit (OTU) method. DADA2 method is more sensitive and specific and can detect real biological variation, which is usually missed by the OTU classification methods. DADA2 can accurately resolve sequence variants differing by just one nucleotide and present in as few as two reads, making this pipeline more precise, comprehensive, and reproducible (35).

Alpha diversity was measured through 5 indices—Observed, Chao1, Shannon, Simpson, and Fisher—to determine the richness and the diversity of the bacteria in the colon mucosa according to the different dietary treatments. A rarefaction curve plot of the number of species (species richness) was plotted as a function of the number of samples (sequence sample size).

### Statistical Analysis

Statistical tests for sequencing analysis were done based on alpha diversity. Statistical tests were performed in R Studio version 3.6.2 by using the following packages:

a. phyloseq (https://www.bioconductor.org/packages/release/bioc/html/phyloseq.html),
b. vegan (https://cran.r-project.org/web/packages/vegan/index.html), and
c. Venn Diagram (https://cran.rproject.org/web/packages/VennDiagram/index.html).

## Results

### Taxonomic Composition

**Figure 2:**
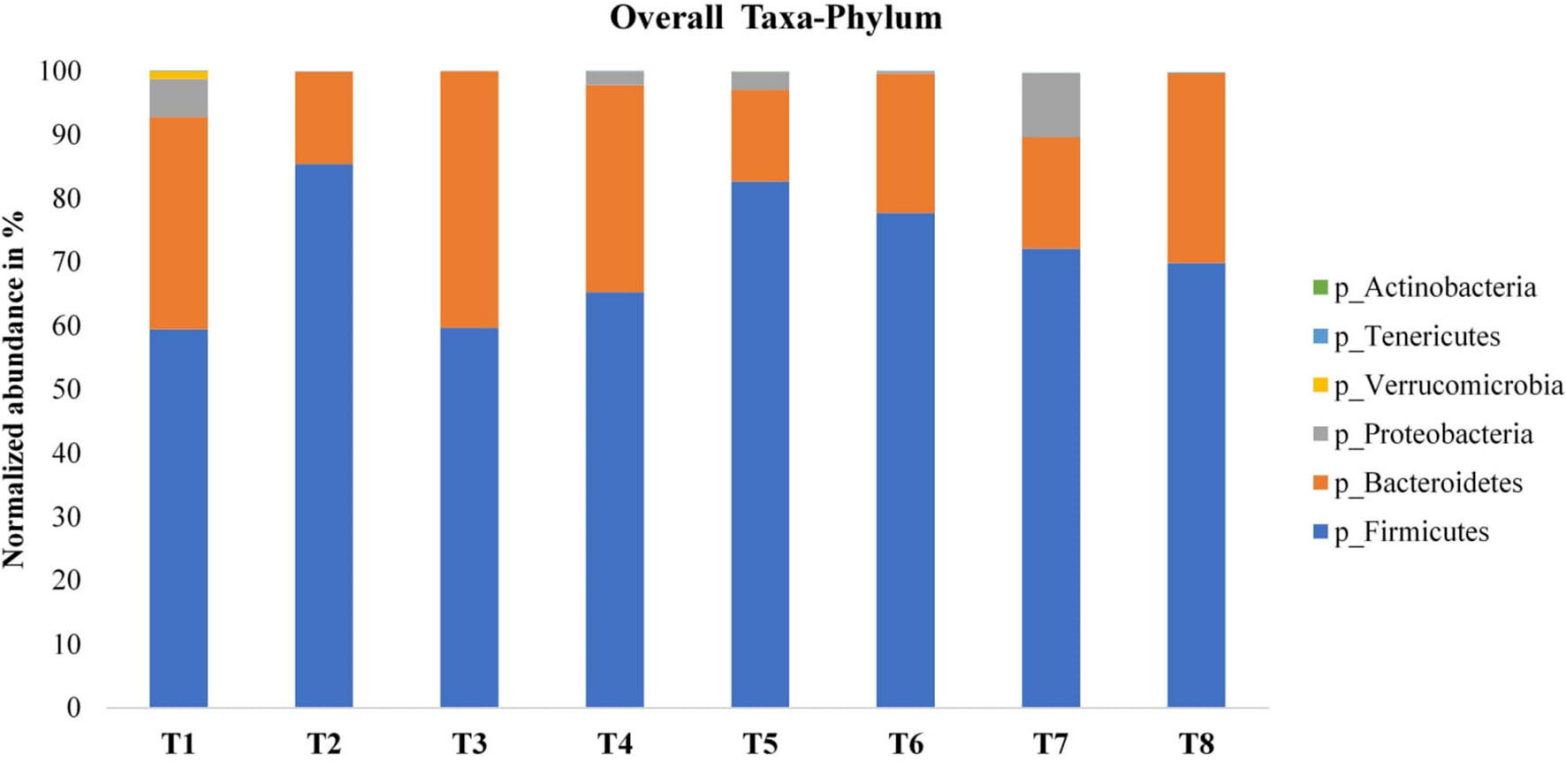
Relative abundance in % of colon microbiota at the phylum level. T1 = Negative control (Basal diet), T2 = Positive control [Basalt diet + 0.01% (w/w) Oxytetracycline], T3 = Basal diet + 0.2% (v/w) postbiotic TL1, T4 = Basal diet + 0.2% (v/w) Postbiotic RS5, T5 = Basal diet + 0.2%(v/w) paraprobiotic RG11, T6 = Basal diet + 0.2% (v/w) postbiotic RI11, T7 = Basal diet + 0.2% (v/w) paraprobiotic RG14, T8 = Basal diet + 0.2% (v/w) paraprobiotic RI11. The most predominant phyla are *Firmicutes* (85.41%) in T2, *Bacteroidetes* (40.24%) in T3, *Proteobacteria* (10.03%) T7, *Verrucomicrobia* 1.21% T1, Tenericutes 0.11% in T8 and *Actinobacteria* 0.06% in T7.

**Figure 3:**
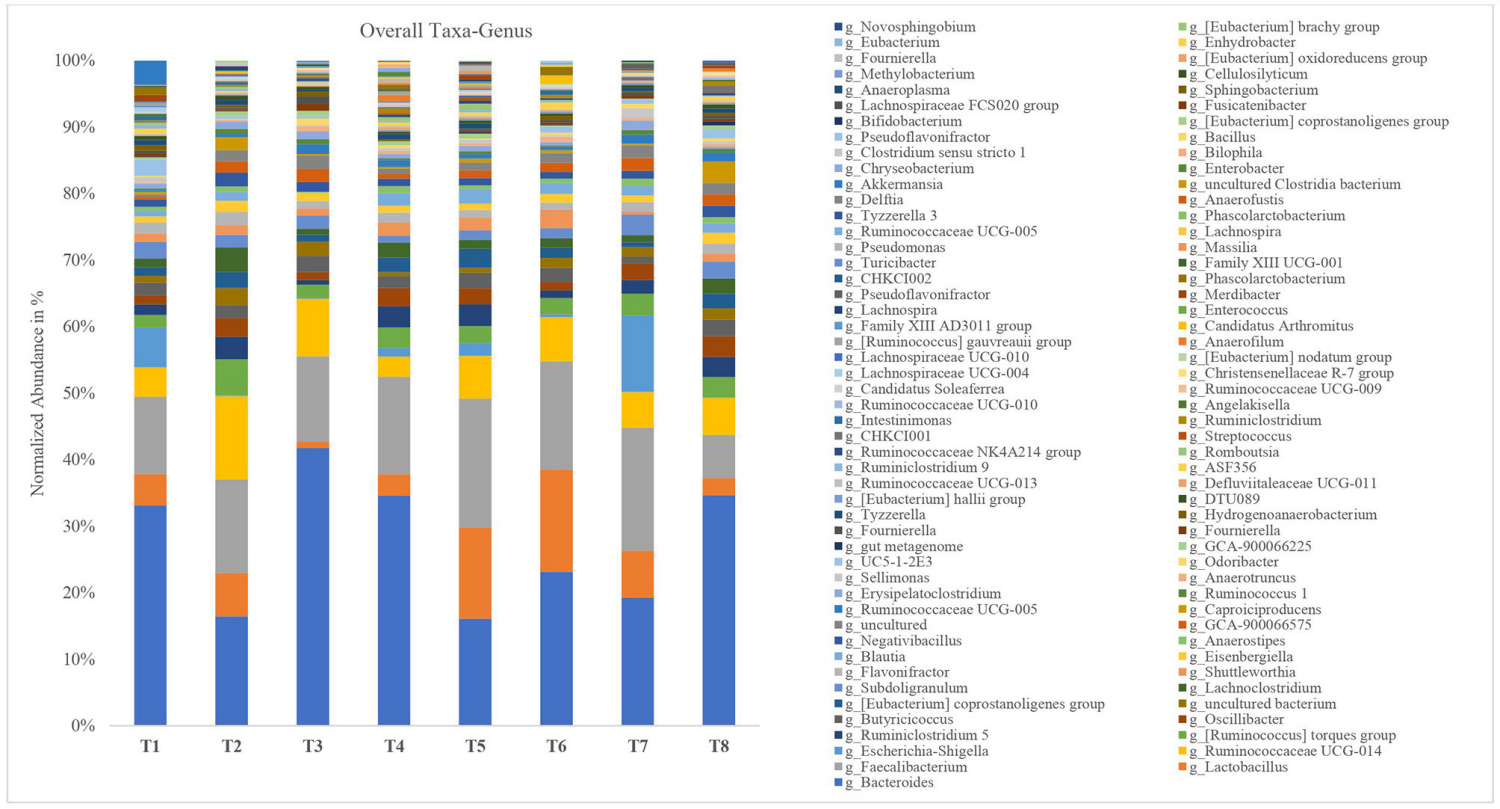
Relative abundance in % of colon microbiota at the genus level. T1 = Negative control (Basal diet), T2 = Positive control [Basalt diet + 0.01% (w/w) Oxytetracycline], T3 = Basal diet + 0.2% (v/w) postbiotic TL1, T4 = Basal diet + 0.2% (v/w) Postbiotic RS5, T5 = Basal diet + 0.2%(v/w) paraprobiotic RG11, T6 = Basal diet + 0.2% (v/w) postbiotic RI11, T7 = Basal diet + 0.2% (v/w) paraprobiotic RG14, T8 = Basal diet + 0.2% (v/w) paraprobiotic RI11. *Bacteroides* (39.36%) in T3, *Faecalibacterium* (17.35%) in T5, *Lactobacillus* (14.39%) in T6, *Ruminococcaceae* UCG-14 (11.01%) in T2, *Escherichia-Shigella* (10.33%) in T7 are the most dominant genus.

The 16S rRNA gene sequencing is a rapid and accurate identification method for bacterial isolates; however, it is not applicable for several genera and only provides resolution till the genus level and the presence of nucleotide variations in rRNA operons in a single genome. Under this category, species distribution under the classification level of phylum up to the classification level of the genus was done. The distribution histogram of the relative abundance was generated as shown in Figures 2, 3. The DADA2 pipeline of colon mucosa samples was classified into six bacterial phyla. Overall, *Firmicutes* (85.41%) in T2, *Bacteroidetes* (40.24%) in T3, and *Proteobacteria* (10.03%) in T7 were the three most dominant phyla (Figure 2). The overall genus showed that *Bacteroides* (39.37%) in T3, *Faecalibacterium* (17.35%) in T5, *Lactobacillus* (14.39%) in T6, *Ruminococcaceae* UCG-14 (11.01%) in T2, *Escherichia-Shigella* (10.33%) in T7, and (*Ruminococcus*) torques group (4.82%) in T2 were the dominant genera (Figure 3).

### Species Diversity

#### Alpha Diversity Indices of Colon Mucosa Microbiota

**Figure 4:**
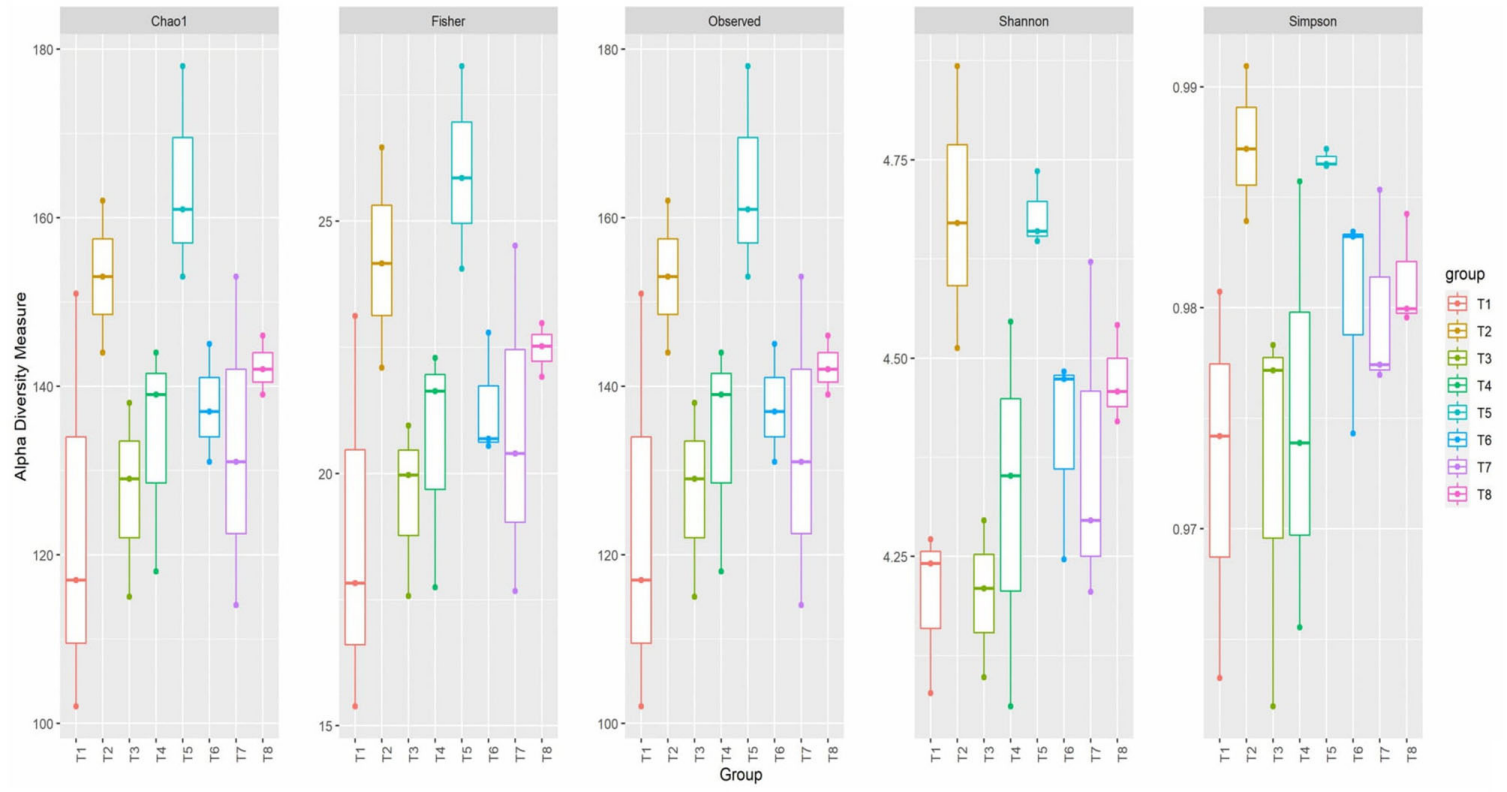
Alpha diversity indices of colon mucosa microbiota of broiler chickens fed postbiotics and paraprobiotics. T1 = Negative control (Basal diet), T2 = Positive control [Basalt diet + 0.01% (w/w) Oxytetracycline], T3 = Basal diet + 0.2% (v/w) postbiotic TL1, T4 = Basal diet + 0.2% (v/w) Postbiotic RS5, T5 = Basal diet + 0.2%(v/w) paraprobiotic RG11, T6 = Basal diet + 0.2% (v/w) postbiotic RI11, T7 = Basal diet + 0.2% (v/w) paraprobiotic RG14, T8 = Basal diet + 0.2% (v/w) paraprobiotic RI11.

The alpha diversity of colon mucosa microbiota of broiler chickens fed postbiotics and paraprobiotics was measured through five indices: Observed, Chao1, Shannon, Simpson, and Fisher (Figure 4). The Observed and Chao1 indices showed increases in richness in the paraprobiotics, positive control, and postbiotics groups. Paraprobiotic RG11 (T5) was recorded to have a higher richness mean value of above 160 ASVs according to the Chao1 index. Fisher's index showed higher species richness in the paraprobiotics, positive control, and postbiotics groups. According to Fisher's index, paraprobiotic RG11 (T5) had the highest richness mean value above 25 ASVs. Shannon's index showed more species diversity in T2 and T5 than the other treatment groups. The positive control (T2) and T5 were recorded to have higher species diversity of above 4.5 on Shannon's index. Similarly, the positive control (T2) and T5 had a higher value on Simpson's index at above 0.985.

**Figure 5:**
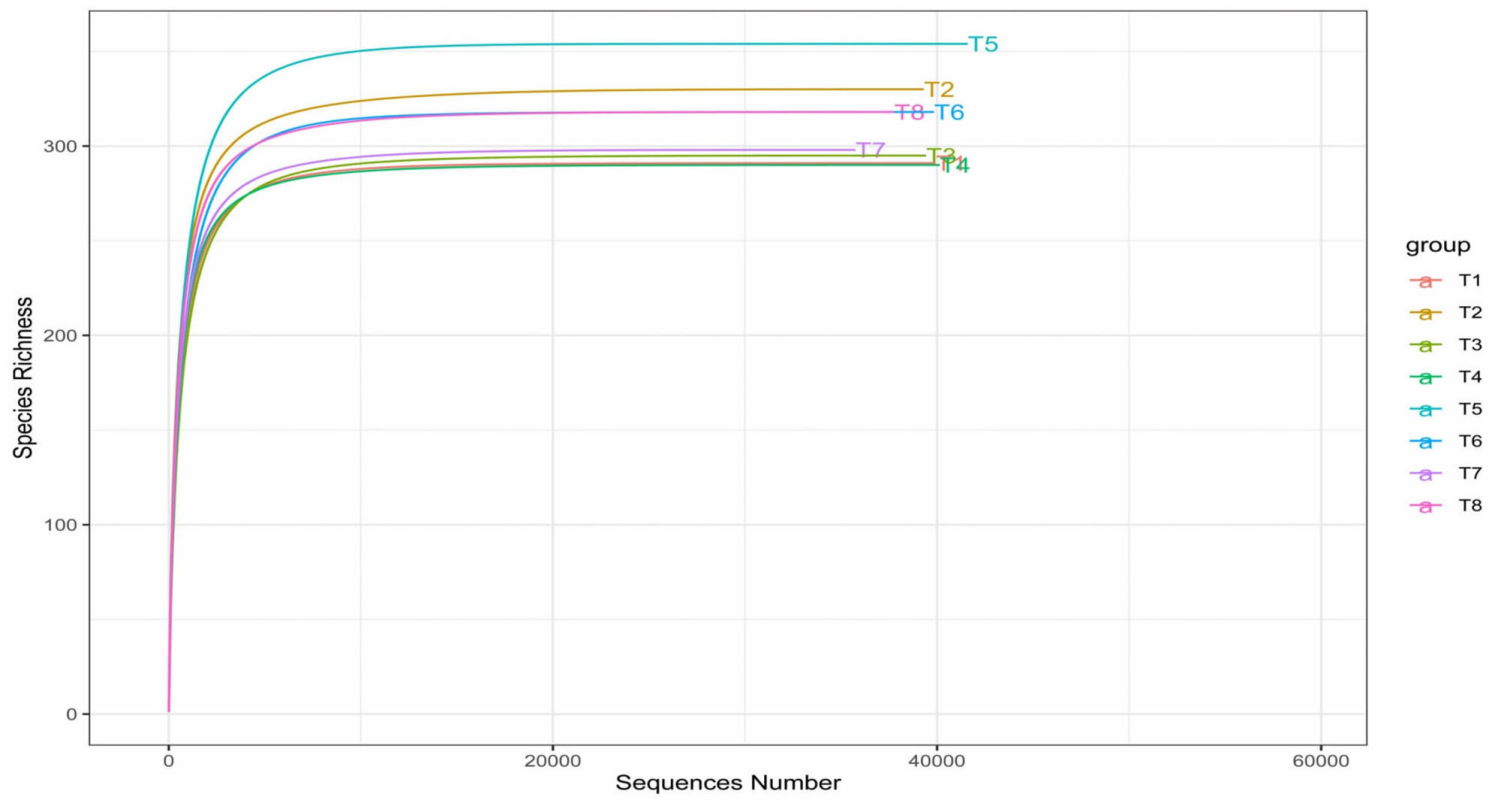
Rarefaction curves of alpha diversity of colon mucosa microbiota. T1 = Negative control (Basal diet), T2 = Positive control [Basalt diet + 0.01% (w/w) Oxytetracycline], T3 = Basal diet + 0.2% (v/w) postbiotic TL1, T4 = Basal diet + 0.2% (v/w) Postbiotic RS5, T5 = Basal diet + 0.2% (v/w) paraprobiotic RG11, T6 = Basal diet + 0.2% (v/w) postbiotic RI11, T7 = Basal diet + 0.2% (v/w) paraprobiotic RG14, T8 = Basal diet + 0.2% (v/w) paraprobiotic RI11. T5 had the highest species richness above 300 detected little above 40,000 sequencing read depth.

The sample-based rarefaction curve in Figure 5 matched the previously determined maximum species richness by Chao1 and Fisher of alpha diversity. Paraprobiotic RG11 (T5) still maintained the highest richness based on the rarefaction curve. According to the rarefaction curves, all the curves of the treatment group reached their plateau, indicating that the read depth was sufficient and less new (new species) can be detected with increasing sequencing depth.

#### Shared and Unique Microbial Composition

**Figure 6:**
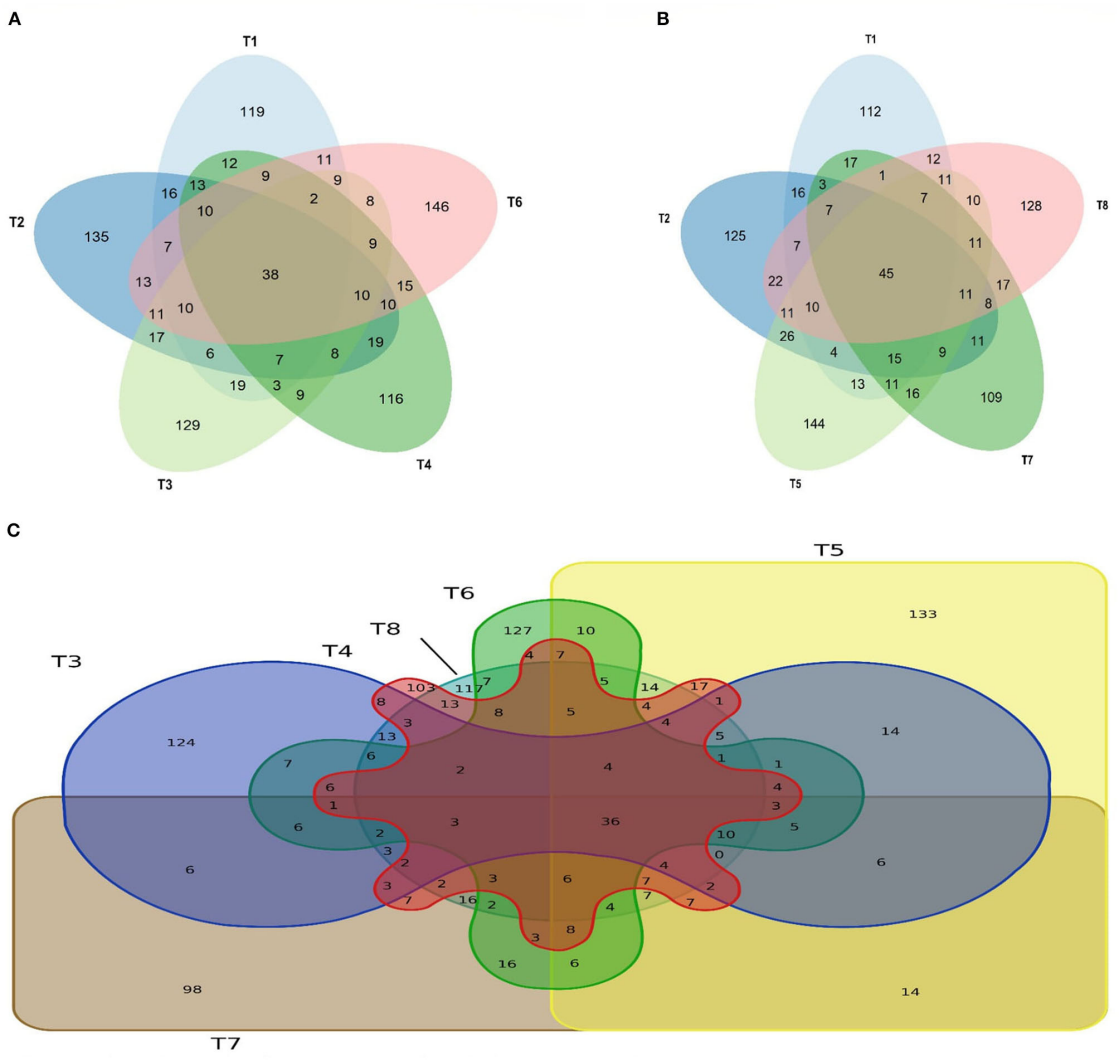
Venn diagram **(A)** species richness of controls and postbiotic group, **(B)** species richness of controls and paraprobiotic group. T1 = Negative control (Basal diet), T2 = Positive control [Basalt diet + 0.01% (w/w) Oxytetracycline], T3 = Basal diet + 0.2% (v/w) postbiotic TL1, T4 = Basal diet + 0.2% (v/w) Postbiotic RS5, T5 = Basal diet + 0.2% (v/w) paraprobiotic RG11, T6 = Basal diet + 0.2% (v/w) postbiotic RI11, T7 = Basal diet + 0.2% (v/w) paraprobiotic RG14, T8 = Basal diet + 0.2% (v/w) paraprobiotic RI11. The highest ASVs among the postbiotics group and the controls **(A)** is 146 T6 and the common number of ASV to them is 38. T5 has 144 ASV as the highest among the paraprobiotics group and the controls **(B)** with 45 ASVs common to them all. Venn diagram **(C)** of species richness of postbiotics and paraprobiotics. T3 = Basal diet + 0.2% (v/w) postbiotic TL1, T4 = Basal diet + 0.2% (v/w) Postbiotic RS5, T5 = Basal diet + 0.2%(v/w) paraprobiotic RG11, T6 = Basal diet + 0.2% (v/w) postbiotic RI11, T7 = Basal diet + 0.2% (v/w) paraprobiotic RG14, T8 = Basal diet + 0.2% (v/w) paraprobiotic RI11. T5 had the highest number of ASV (133) and only 36 ASVs are common postbiotics and paraprobiotics.

The comparison of the control and postbiotics groups showed that 38 ASVs were common to the five treatment groups, and 146 more unique ASVs were found in T6 (Figure 6A). On the other hand, the comparison between the control groups and paraprobiotic groups showed that a total of 45 ASVs were common to the five treatment groups, and 144 more unique ASVs were found in T5 (Figure 6B). The Venn diagram in Figure 6C represents the comparison of ASVs between the postbiotics and paraprobiotics groups. The results showed that out of the total abundance ASVs found in the six treatments, T5 had 133 more unique ASVs, and 36 ASVs were common to both the postbiotics and paraprobiotics groups.

#### Phylogenetic Tree

**Figure 7:**
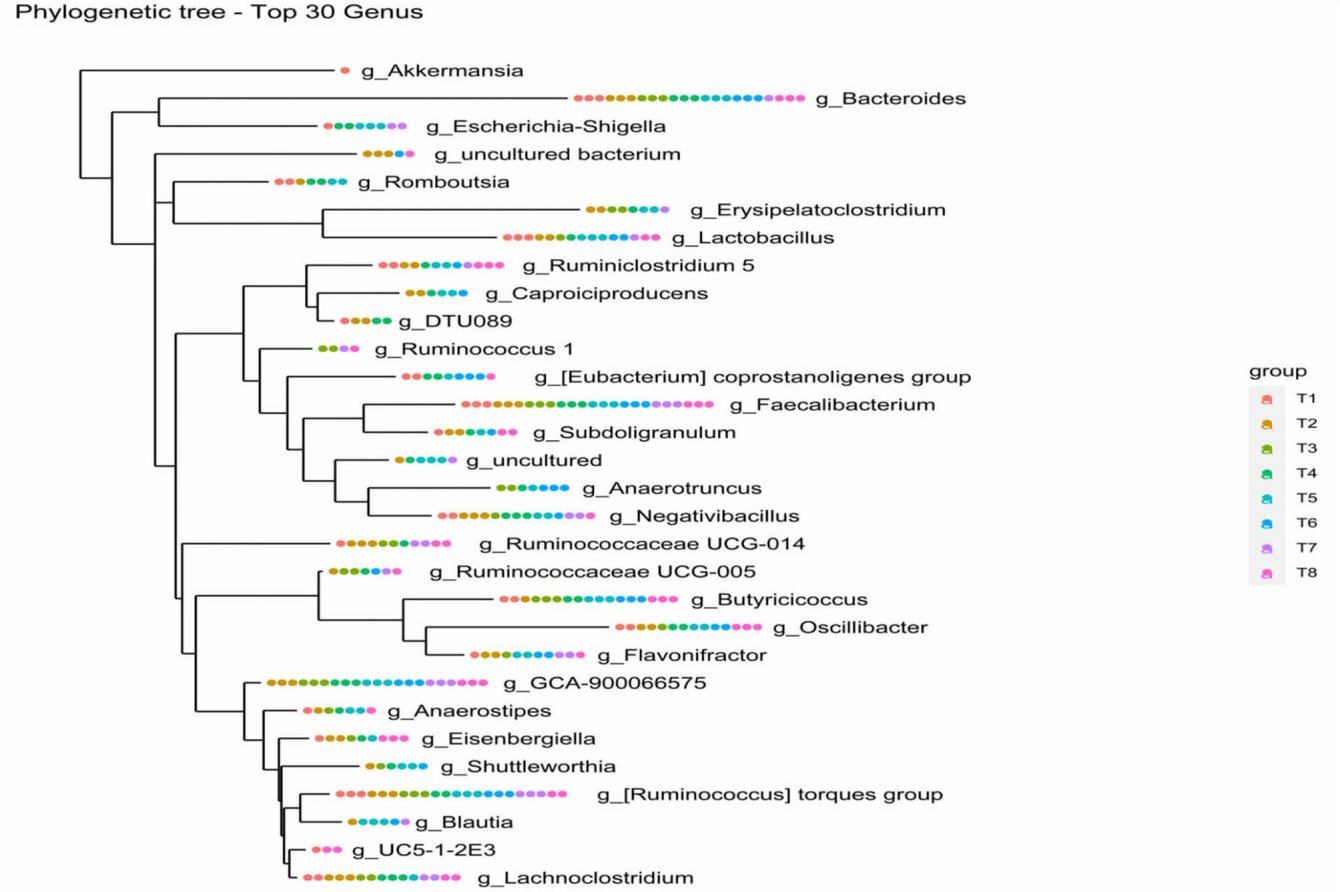
Phylogenetic tree of top 30 genus of colon mucosa microbiota. T1 = Negative control (Basal diet), T2 = Positive control [Basalt diet + 0.01% (w/w) Oxytetracycline], T3 = Basal diet + 0.2% (v/w) postbiotic TL1, T4 = Basal diet + 0.2% (v/w) Postbiotic RS5, T5 = Basal diet+ 0.2%(v/w) paraprobiotic RG11, T6 = Basal diet+0.2% (v/w) postbiotic RI11, T7 = Basal diet + 0.2% (v/w) paraprobiotic RG14, T8 = Basal diet + 0.2% (v/w) paraprobiotic RI11. The genus Bacteroides was found in all the treatments, and the overall highest closely related genus.

The relationship between the bacterial species was studied by constructing a phylogenetic tree using a combination of MAFFT and FastTree algorithms based on maximum-likelihood NNIs. The phylogenetic tree confirms the genus to which the query sequence strain belongs and its closest neighbours by comparing it with other sequences from the database. Further, genotypic, chemotaxonomic, and phenotypic analysis platforms are designed. The overall most closely related genus among the 30 top genera according to ASVs in colon mucosa microbiota of the eight dietary treatments was the genus *Bacteroides* (see Figure 7).

## Discussion

### Effect of Postbiotics and Paraprobiotics on 16S RRNA Amplicon Sequencing of Colon Mucosa Microbiota

According to (36), the GIT of chickens harbours a large microbial community that plays an important role in chicken growth and health through enhancing nutrient absorption and strengthening the immune system. The GIT provides a platform for the growth of a diverse microbiota that provides a second barrier against colonisation by pathogens, regulates immune development and maturation, and provides metabolites for host nutrition (37–39). SCFA, such as butyrate, provides energy for the epithelial cells that line the intestine and subdues the expression of virulence factors of harmful (38, 40–42).

There is no doubt that caecal microbiota has the greatest diversity in the GI tract. It is the source for most, if not all, mucosa-associated microbiota of the proximal and distal colon (43). Until now, most studies on chicken intestinal microbiota are focused more on the caecal section of the hindgut. However, using 16S rRNA amplicon sequence, we focused the current research on the colon mucosa microbiota of broiler chickens.

The six most abundant phyla reported in this study are Firmicutes, Bacteroidetes, Proteobacteria, Verrucomicrobia, Tenericutes, and Actinobacteria in the colon mucosa. Similarly, the five most abundant phyla reported in the duodenum, jejunum, ileum, colon, and caecum microbiota were Firmicutes, Bacteroidetes, Proteobacteria, Actinobacteria, and Cyanobacteria (44); this elucidated the fact that caecal microbiota was the major source of mucosa-associated microbiota of the distal colon (43). According to our findings, Firmicutes was the dominant phylum in all the treatment groups with a significantly higher abundance of 85.41% in T2 and 82.66% in T5 than other treatments. The alpha diversity indices (Chao1, Fisher, Observed, Shannon, and Simpson) indicated an increase in species diversity and richness in the colon mucosa microbiota.

The implication of the increase in abundance of Firmicutes as a result of dietary supplementation with postbiotics and paraprobiotics in this study is more butyrate production. Firmicutes were reported as the phylum with a larger number of taxa encoding enzymes required for butyrate production (45). Previously, postbiotic metabolite combination was reported to increase the faecal butyric acid concentrations in broiler chickens (14). Furthermore, butyrate is the main source of energy for enterocytes, and it helps regulate cellular differentiation and proliferation within the intestinal mucosa, thereby increasing intestinal tissue weight (40, 46–48). The stimulation of the release of gastrointestinal peptides and growth factors by butyrate acting on cell proliferation is one of the major mechanisms involved in intestinal mucosa proliferation by butyrate (46). In addition, previous research findings revealed that butyrate increases the secretion of IL-10 and decreases the secretion of interferon-g by activated human lymphocytes *in vitro* (49, 50). Also, there was a reported decrease in *ex vivo* production of inflammatory cytokines in intestinal biopsies of humans who have Crohn's disease and a reduction in the severity of 2,4,6-trinitrobenzene sulfonic acid-induced colitis in rats caused by butyrate (51).

*Bacteroides* (39.37%), *Faecalibacterium* (17.35%), *Lactobacillus* (14.39%), *Ruminococcaceae* UCG-014 (11.01%), and *Escherichia-Shigella* (10.33%) were the most dominant out of the overall genera sequenced in this study. Similarly, it was reported that *Streptococcus*, uncultured *Ruminococcaceae*, and *Lactobacillus* were the three most predominant genera in the colonic digesta and mucosa of pigs (52). The production of propionate and succinate was associated with *Bacteroides* as terminal products of metabolism, as reported by Adamberg et al. (53). Propionate is a less preferred substrate of colonocytes but is transported to the liver and used as an important energy source for the host (47). The strain *Faecalibacterium* was reported to be a carrier of the enzymes necessary for butyrate production and present from the early stages of development. Therefore, the strain will actively participate in future intervention and modulation of the gut microbiota by improving the overall health and growth performance of poultry (45). It was revealed recently that some *Ruminococcus* species in the human colon were found to play a primary role in the degradation of dietary resistant starch (54). *Lactobacillus*, an important probiotic bacterium in promoting a healthy gut, was the fourth most predominant genus. A recent study with postbiotics also revealed a significant (*p* < 0.05) increase in the population of *Lactobacillus* in the caecum of broiler chickens (17). *Lactobacillus* is a beneficial microbe that can produce bacteriocins, a natural antimicrobial compound capable of inhibiting the growth of pathogens at molecular and cellular levels (55). The presence of *Lactobacillus* could explain why the population of chicken pathogens was significantly inhibited in this study. The phylum Proteobacteria in the colon mucosa was greatly decreased by postbiotics, paraprobiotics, and positive control, except for paraprobiotic RG14 (T7), where its population was higher.

## Conclusion

This study revealed that supplementation of postbiotics and paraprobiotics in the broilers' chicken diet demonstrated a positive effect on the microbiota by supporting the increase of beneficial microbes like the Firmicutes while decreasing harmful microbes like the Proteobacteria. The modification in the microbiota can result in a healthier gut. Therefore, postbiotics and paraprobiotics can positively affect the microbiota of the colon mucosa.

## Data Availability Statement

The original contributions presented in the study are included in the article/supplementary material, further inquiries can be directed to the corresponding author/s.

## Ethics Statement

The animal study was reviewed and approved by Institutional Animal Care and Use Committee (IACUC) with a Reference No: UPM/IACUC/AUP-R098/2018.

## Author Contributions

YD, TCL, and HLF designed the study. HLF and TCL provided probiotic strains and methods to produce postbiotics, probiotic suspension, and paraprobiotics and modified the manuscript. YD and AMN helped to perform the experiments. YD analysed the data. HA and NAMT assisted in proofreading the manuscript. All authors contributed to the article and approved the submitted version.

## Conflict of Interest

The authors declare that the research was conducted in the absence of any commercial or financial relationships that could be construed as a potential conflict of interest.

## Publisher's Note

All claims expressed in this article are solely those of the authors and do not necessarily represent those of their affiliated organizations, or those of the publisher, the editors and the reviewers. Any product that may be evaluated in this article, or claim that may be made by its manufacturer, is not guaranteed or endorsed by the publisher.
